# Dublin Correspondence

**Published:** 1867-10

**Authors:** Lunsford P. Yandell

**Affiliations:** Dublin


### ARTICLE IV.

DUBLIN, August 9th, 1867.

*Dr. J. M. Johnson:—*

The British Medical Association met in this Metropolis for the first time on the 6th of August, and rarely, I suppose, has a more remarkable assemblage of medical men ever taken place. The fact that it was the first ever held in Ireland, and that it was presided over by that great ornament of our profession, Dr. William Stokes, imparted an unusual interest to the meeting. I was fortunate enough to bear a letter of introduction to Dr. Stokes from my friend Dr. Lewis Ropes, which insured me an affable reception and free access to the meetings of the Association. I met him at the University buildings on the morning when the Association convened and received from him an invitation to be present at the delivery of his Inaugural Address, at 2 o'clock that afternoon, and he also invited me to call upon him next morning and accompany him to the public breakfast to be given to the members of the Association.

From the reputation Dr. Stokes has long enjoyed in our country you are prepared to hear that he is now a man far advanced in years, though hale still and capable of much profitable labor. He is somewhat under six feet in height, with broad shoulders, much stooped, large, majestic head, fringed with thin iron-gray hair, very long and curling, but nearly bald on top. The expression of his face is very benevolent, and you are gratified to remark in its lineaments the impress of goodness as well as greatness. His features are heavy and not handsome. He is singularly abstracted in manner and gives you the impression that he is profoundly absorbed in thought. In fact, as you gaze upon his calm, thoughtful face the idea occurs to you that it is only the tenement of clay you are beholding, and you wonder where is now the great mind that animates it and in what work engaged. Dr. Stokes is Regius Professor ot Physic in the University of Dublin.

After some preliminary business by the Association and a few remarks by the retiring President, Dr. Waters, of Edinburg, Professor Stokes began his address amid the most enthusiastic applause. This grand old Irish gentlemen stood as unmoved by the demonstration as one of native old oaks would be by the summer breeze, not heeding the clapping of hands, and stamping, and cheering of the four or five hundred doctors before him any more than if they had been so many crickets or grasshoppers. He made no attempt at oratory, but read his discourse in a deliberate manner and in a voice so low that I found it impossible to hear him without the closest attention. The delivery of the address required about an hour, which, as was to have been expected from the great reputation of the author evidenced deep thought, finished scholarship and careful preparation, and was pervaded by a spirit of earnest piety. It was, in every respect, worthy of the mind which has borne so large a part in the advancement of modern medicine.

The British Medical Association is a splendid body of men, and among its members are many names which hold the most conspicuous place in the profession; but I have found both physiognomy and phrenology sadly at fault when I have, by their light, attempted to fix upon its leading intellects.—Experience has taught me that I cannot pick out the giants in a crowd by merely inspecting their countenances and heads, for the great men often look little, and the little men as often look great. Men are like trees, which, whatever may be their stateliness of form and show of foliage and flowers, can only be judged of by their fruits. In assemblies like this it would be a great satisfaction to strangers, if the great men were labelled as the fine pictures are in some galleries.

At the appointed hour on Wednesday morning I accompanied Dr. Stokes to the public breakfast, which was served in an immensely long hall, having two rows of tables, and around these were seated about four hundred doctors. The evidence of their enjoyment was afforded by the activity with which they plied their knives, forks and tongues, for never, I am sure, was greater racket made by a flock of blackbirds or a female tea party, than by this breakfast party of *savans.* I had the honor to sit between the President and Dr. Ackland, the President elect. After breakfast I was introduced to Dr. A. by Pofessor Stokes, and was amused by his remark, "if I had known you were an American, sir, I should have begun a conversation with you at the table, but I thought you was an Italian, and felt too uncertain about my knowledge of that language to attempt to speak it." If he had spoken to me in Italian I am not sure whether the joke would have been more decidedly upon him or upon myself.

Breakfast over, I went, with a number of others, in company with Dr. Stokes to the Royal College of Surgeons, and thence to the University Club, where our names were registered, and we were admitted to all the privileges of membership during our stay in Dublin. We were informed that we might amuse ourselves in reading or writing, or in eating or drinking, in billiards or whist whichever we felt inclined.

From the Club I went with Dr. Ackland, Sir James Y. Simpson, and Mr. Teale on a jaunting car to the Hardwick Hospital, where Dr. Gordon showed us a number of cases of Cerebrospinal Meningitis. He called our attention to the fact, that most of the cases in the present epidemic are adults and persons well advanced in life, instead of children who were formerly its favorite victims. At 11 o'clock the Association met, but I am sorry to say that I was prevented, by indisposition, from attending the meeting.

On Thursday I had the good fortune to hear Dr. Pirrie's interesting and valuable paper on *Acupressure*, and an exceedingly interesting discussion which followed on this important subject. On Friday I heard a very able paper by Sir James Y. Simpson on the Cephalotribe, and some extended remarks by the same gentleman on the use of cloroform. Acupresure, it is contended by Prof. Pirrie, is far superior to the ligature in arresting hemorrhage resulting from surgical operations, as the ligature is to the ancient practice of searing with a hot iron. It is attended by no loss of blood, no supperation, and instead of the daily dressings for weeks, under the old method of ligatures, the wound heals without any dressings, in a few days. The acupressure is accomplished in the twinkling of an eye, and the operator requires no assistant, as he does when the vessels are to be ligated. Sir J. Y. Simpson, the originator of the improvement, I believe, is a devoted and enthusiastic advocate of the process; and, in fact, I am safe in stating that the voice of the British Medical Association is emphatically in favor of this simple but wonderful surgical operation.

The application of Carbolic acid to the cut surfaces in surgical operations was also discussed at this meeting. In Germany, where the practice originated, it has lost its hold upon the confidence of the professiou and is now seldom resorted to. The verdict of the Association is that it is safer to let the acid alone.

In reply to a question as to the relative merits of Cephalotripsy and the Caesarian section, Professor Simpson remarked, that were the child dead he should have no hesitation about resorting to the cephalotribe, but that if the child was living he should hesitate a long time before using this instrument, for it was a grave matter to decide upon the taking of human life. He rather inclined to the belief that if a woman with a deformed pelvis would go on putting herself in the way of becoming pregnant, she ought to be made to take the risks of the Ceasarian operation, rather than be encouraged in her course by sacrificing the life of her child.

Prof. Simpson uses chloroform in all cases of labor, whether difficult or simple, laying down the rule, which he insists is a golden one, that it is to be given during the continuance of the pains, and omitted in the intervals. Administered in this way, it hastens the labor, and no evil ever results from its application. It does not predispose to hemorrhage, nor to perpetual convulsions, but on the contrary tends to ward these off, and when they occur is the most efficient remedy for them. In giving it to his patients he employs a simple napkin or towel, having discarded all the various inhalers proposed for its superadministration. He does not measure the quantity, but continues to give it until anasthesia is induced. He insists upon perfect quiet as of vital importance in the lying-in chamber; and he contends, with great reason, that the recumbent posture should always be assumed where chloroform is inhaled, whether by direction of the surgeon, the obstetrician, or the dentist. Going beyond his own department in which his authority is so high, Sir James asserts that chloroform is the best of all remedies for an incipient Catarrh, especially by doctors, who are so averse to taking medicine; and that it is also one of the most efficient of Collyria. In Catarrh, you pour a little of the fluid into the palm of the hand and inhale the vapor, and in ophthalmia you bring the vapor in contact with the eye. Returning to the use of anæsthetics in midwifery, he expressedh imself in words to the following effect: "A man who should whip a poor sick woman with a cat-of-nine tails would be considered exceedingly cruel and would probably be punished by law for his cruelty. The act would merit some punishment, but he rather thought that the accoucheur who permitted his patients to suffer the cruel pangs of childbirth, at this day, was guilty of a sin of omission almost as heinious. So safe has chloroform come to be considered in Edinburg that the nurses and old women administer it, and Sir James usually finds his patients under its influence when he arrives at their bedsides. It is the belief of this sanguine discoverer that in half a century or a hundred years the profession will have learned how to administered all our remedies in the form of vapors. He spoke in high terms of praise of the oil of juniper as a diurstic inhaled in that form. He puts a spoonful of the oil into a vessel of hot water and directs the patient to breath the steam.

Dr. Henry Bennet gives his testimony in favor of chloroform in midwifery. He believes that if it were the law of nature that husbands should take turn about with their wives in bearing children, the men would all at once become violent advocates of anæsthetice. I may remark that chloroform seemed to have no enemies in the British Medical Association. Prof. Simpson asserts that it never stupefies or ot] e wise injures the child, as is sometimes done by Sulphuric ether. This eminent man was the master spirit of the Association, and is, without question, the foremost medical man of all the world. He is the most attractive and amusing speaker to whom I have ever listened on scientific subjects. His language is always chaste, perspicuous and elegant, and he gives you kernels of wisdom coated with sugar, and shows up his stores of knowledge flavored with the most delightful wit and humor. He is a philosopher of the highest order, but a jolly one, who makes you laugh while you learn. I am strongly tempted to give you some of the amusing anecdotes with which he illustrated his opinions, but my letter is already so long that I must abstain. Next to Sir J. Y. Simpson. Prof. Pirrie was the best speaker whom I heard in the Association. Both speak with a broad Scotch accent.

During the four days of the meeting there were daily breakfasts, dinners, lunches and conversaziones given to the members, and the great event of the week in Dublin was the assembling of this body of distinguished men, whose labors are acknowledged to be intimately connected with human happiness and social progress. The papers and discussions before the Association afforded gratifying evidence to all of the high state of development to which all the branches of therapetics have attained. Never in the history of the healing art was there probably ever before so great a concentration upon the science of intellectual power, rendition and experience as was represented at this meeting and as is engaged in the advancement of medicine all over the world. And while this is a matter of congratulation to men everywhere, it was to Irishmen a cause of especial pride that their own physicians occupied so eminent a position at this meeting, and are regarded as among the most successful cultivators ot Medical science.

On Monday I go to Paris, to be present at the meeting of the International Medical Congress, which takes place on the 15th instant.

LUNSFORD P. YANDELL, JR.

